# Proteomics analysis of FUS mutant human motoneurons reveals altered regulation of cytoskeleton and other ALS-linked proteins via 3′UTR binding

**DOI:** 10.1038/s41598-020-68794-6

**Published:** 2020-07-16

**Authors:** Maria Giovanna Garone, Vincenzo Alfano, Beatrice Salvatori, Clarissa Braccia, Giovanna Peruzzi, Alessio Colantoni, Irene Bozzoni, Andrea Armirotti, Alessandro Rosa

**Affiliations:** 1 Department of Biology and Biotechnology Charles Darwin, Sapienza University of Rome, P.le A. Moro 5, 00185 Rome, Italy; 2 Center for Life Nano Science, Istituto Italiano Di Tecnologia, Viale Regina Elena 291, 00161 Rome, Italy; 3 D3 PharmaChemistry, Istituto Italiano Di Tecnologia, Via Morego 30, 16163 Genoa, Italy; 4 Analytical Chemistry Lab, Istituto Italiano Di Tecnologia, via Morego 30, 16163 Genoa, Italy; 5 Laboratory Affiliated to Istituto Pasteur Italia-Fondazione Cenci Bolognetti, Department of Biology and Biotechnology Charles Darwin, Sapienza University of Rome, Viale Regina Elena 291, 00161 Rome, Italy; 6 Present Address: UMR INSERM 1052-CNRS, Cancer Research Center of Lyon (CRCL), 5286 151 cours Albert Thomas, 69424 Lyon Cedex 03, France

**Keywords:** Amyotrophic lateral sclerosis, Induced pluripotent stem cells, Mass spectrometry, Disease model, Motor neuron

## Abstract

Increasing evidence suggests that in Amyotrophic Lateral Sclerosis (ALS) mutated RNA binding proteins acquire aberrant functions, leading to altered RNA metabolism with significant impact on encoded protein levels. Here, by taking advantage of a human induced pluripotent stem cell-based model, we aimed to gain insights on the impact of ALS mutant FUS on the motoneuron proteome. Label-free proteomics analysis by mass-spectrometry revealed upregulation of proteins involved in catabolic processes and oxidation–reduction, and downregulation of cytoskeletal proteins and factors directing neuron projection. Mechanistically, proteome alteration does not correlate with transcriptome changes. Rather, we observed a strong correlation with selective binding of mutant FUS to target mRNAs in their 3′UTR. Novel validated targets, selectively bound by mutant FUS, include genes previously involved in familial or sporadic ALS, such as *VCP*, and regulators of membrane trafficking and cytoskeleton remodeling, such as *ASAP1*. These findings unveil a novel mechanism by which mutant FUS might intersect other pathogenic pathways in ALS patients’ motoneurons.

## Introduction

The motoneuron disease Amyotrophic Lateral Sclerosis (ALS) has been linked to mutations in several RNA binding proteins (RBPs) and altered RNA metabolism^1,2^. ALS mutations affecting nuclear localization of the RBP FUS are regarded as a primary event, eventually leading to motoneuron death by unknown mechanisms^3,4^. Accordingly, FUS mutations that cause higher levels of mislocalization in the cytoplasm (such as the P525L) are associated to more aggressive and juvenile ALS pathology^5^. However, little is known about the effects of abnormal FUS accumulation in the cytoplasm on its RNA targets. A change in the translatome has been previously observed in neurons derived from mouse embryonic stem cells ectopically overexpressing human FUS-R495X protein, which is another mutant FUS mislocalized to the cytoplasm^6^. Alteration of protein translation was also studied upon oxidative stress and ectopic expression of wild-type (WT) or R495X mutant FUS in neuroblastoma cells^7^. However, overexpression of WT FUS at non-physiological levels is known to produce toxic effects per se^8^. Moreover, since ALS specifically affects motoneurons^9^, disease-relevant targets might be missed in in vitro models based on non-motoneuronal cells. The effect of mutant FUS on the proteome of human motoneurons remains unexplored.

We have recently developed an in vitro cellular model consisting of isogenic pairs of mutant (P525L) and WT FUS human induced pluripotent stem cell (hiPSC) lines^10^. These cells can be differentiated into ventral spinal cord populations and sorted according to a *Hb9::*GFP reporter, providing pure samples of human motoneurons^11^. Using this cell model, we have previously identified alterations in the human motoneuron transcriptome and defined the RNA interactome of WT and mutant FUS^11,12^. Notably, FUS^WT^ is mostly associated to intronic regions whereas FUS^P525L^ preferentially binds 3′UTRs^12^. For the transcript of neural RBP ELAVL4/HuD, mutant FUS binding to the 3′UTR leads to increased protein levels^12^. Here, we performed proteomics analysis to assess the broad effects of the altered binding of mutant FUS. By cross-checking proteomics, transcriptomics and interactomics data, we gained insights into the molecular mechanisms underlying deregulation of selected targets.

## Results

### Proteome alteration in FUS mutant motoneurons

**Figure 1:**
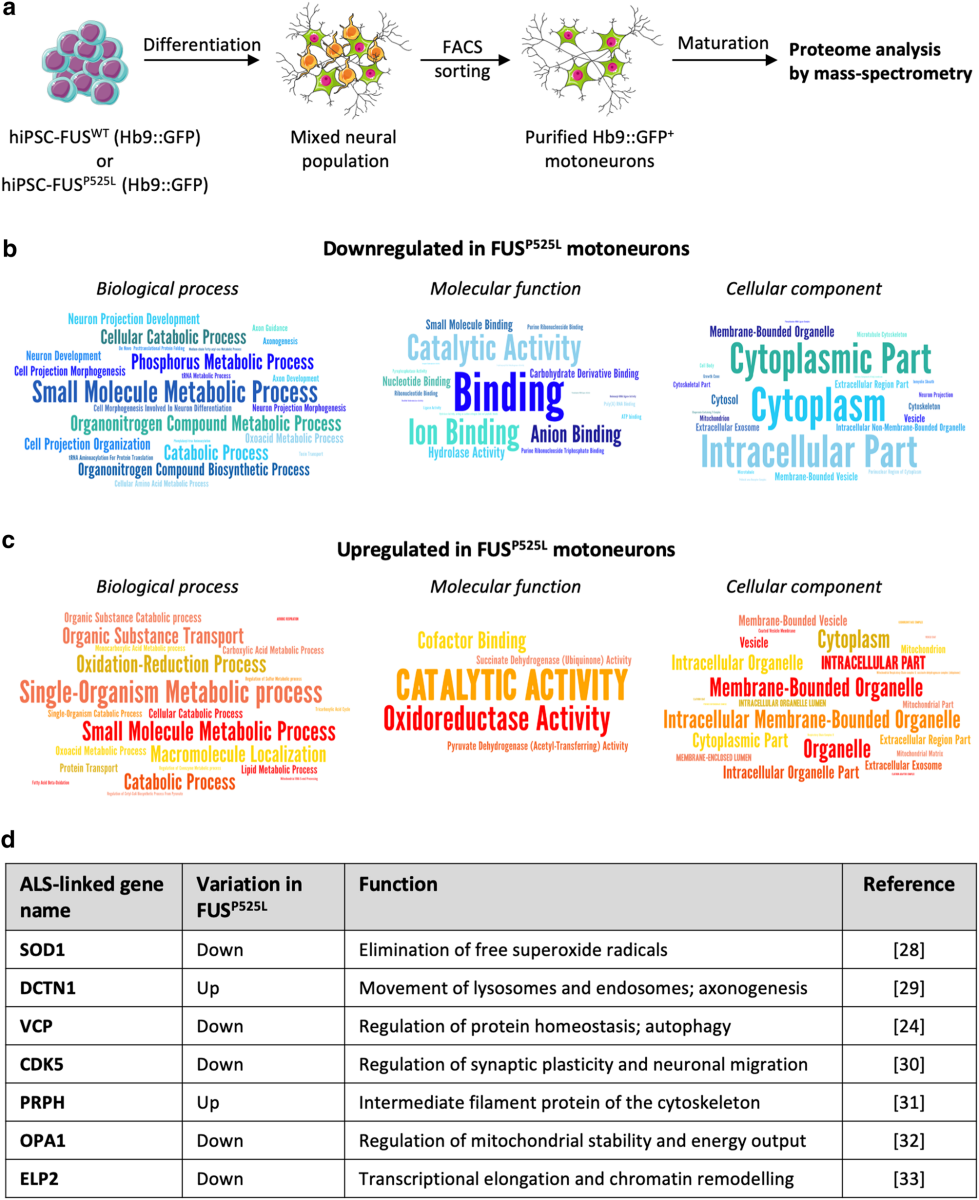
Mass-spectrometry analysis in FUS^WT^ and FUS^P525L^ motoneurons. (**a**) Outline of the generation of pure motoneuron samples from isogenic FUS^WT^ and FUS^P525L^ hiPSC lines. An *Hb9*::GFP reporter was used for isolation of motoneuron progenitors by FACS. After subsequent maturation, protein samples from four independent differentiation experiments were collected for proteome analysis by mass-spectrometry. This figure was drawn using the vector image bank of Servier Medical Art (https://smart.servier.com/). Servier Medical Art by Servier is licensed under a Creative Commons Attribution 3.0 Unported License. (**b**, **c**) Word clouds generated by FIDEA (https://omictools.com/fidea-tool)^34^ representing GO “Biological Process”, “Molecular Function” and “Cellular Component” terms enriched in the set of proteins that are downregulated (**b**) or upregulated (**c**) in FUS^P525L^ motoneurons. The categories are represented with a character size proportional to the statistical significance of their enrichment. The images have been taken by Maria Giovanna Garone. (**d**) Table showing ALS-linked genes,^24,28–33^ from the DISEASES web resource^14^, encoding for proteins that are significantly up- or downregulated in FUS^P525L^ motoneurons.

Human motoneurons were obtained by differentiation of isogenic FUS^WT^ and FUS^P525L^ (carrying the homozygous P525L mutation) hiPSCs^10^. Motoneuron progenitors were sorted according to the expression of a *Hb9*::GFP reporter stably inserted in the *AAVS1* locus giving rise, upon further maturation, to homogenous populations of cells with neuronal morphology^11^ (Supplementary Fig. S1 online). These cells were used for label-free proteomics analysis by mass-spectrometry (high-resolution liquid chromatography with tandem mass spectrometry, LC–MS/MS) (Fig. 1a). Protein quantification was performed in SWATH (Sequential Windowed Acquisition of All Theoretical Fragment Ion Mass Spectra) mode using a library of more than 10,500 human proteins (Pan Human Ion Library)^13^, representing about half of the proteins annotated in the UNIPROT human reference proteome. A principal component analysis (PCA) plot showing clustering of FUS^WT^ and FUS^P525L^ motoneurons samples is shown in Supplementary Fig. S1 online. We identified 323 proteins differentially expressed in FUS^WT^ and FUS^P525L^ motoneurons at *p* value < 0.05 (Supplementary Fig. S1 online; Supplementary Table S1 online). We then performed gene ontology (GO) term enrichment analysis on proteins that were downregulated (169) and upregulated (154) in FUS mutant cells (Supplementary Table S2 online). In the downregulated group we noticed categories related to neuron development, differentiation and morphogenesis, and in particular to metabolic processes and neuron projection (Fig. 1b, left), and terms related to cytoplasm and cytoskeleton (Fig. 1b, right). Analysis of upregulated proteins revealed categories related to catabolic processes and oxidation–reduction (Fig. 1c). We then interrogated the DISEASES web resource^14^ and crossed the list of differentially expressed proteins with a list of ALS-associated genes from manually curated literature. As shown in Fig. 1d, 2 upregulated and 5 downregulated proteins have been previously linked to ALS.

**Figure 2:**
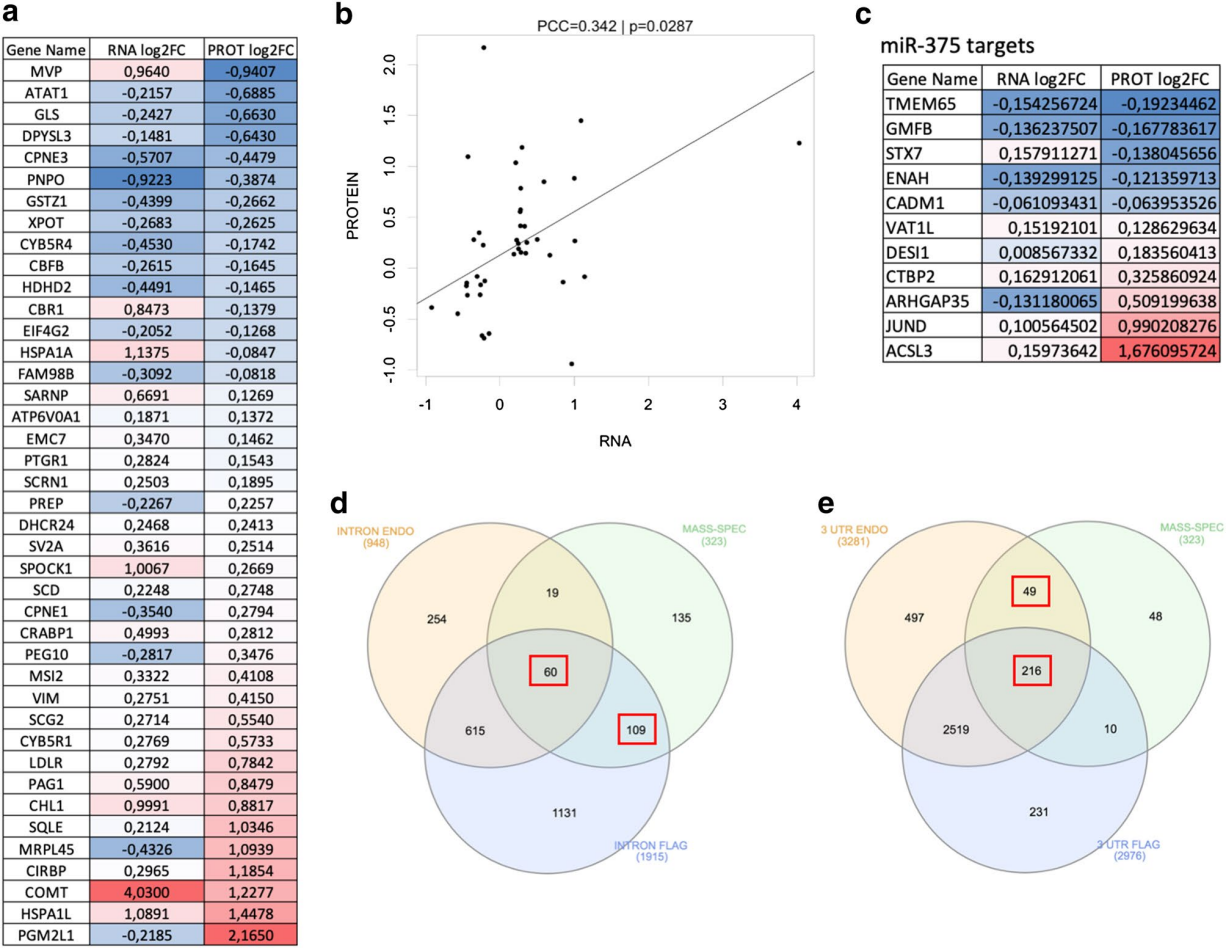
Proteomics, transcriptomics and FUS interactomics analyses in motoneurons. (**a**) Table showing genes encoding for differentially expressed proteins, ordered by fold change, and differentially expressed mRNAs, in FUS^P525L^ motoneurons compared to FUS^WT^ ones. Color code: blue, downregulated; red, upregulated. (**b**) Correlation between transcript and protein levels of genes enlisted in (**a**). PCC: Pearson correlation coefficient; p: *p* value. (**c**) Table showing miR-375 target genes that encode for differentially expressed proteins in FUS^P525L^ motoneurons. Color code: blue, downregulated; red, upregulated. (**d**) Venn diagram showing the overlap between proteins that are altered, in any direction, in FUS^P525L^ motoneurons (“MASS-SPEC”) and transcripts that are bound in intronic regions by endogenous FUS^WT^ (“INTRON ENDO”) or exogenous FLAG-FUS^WT^ (“INTRON FLAG”). (**e**) Venn diagram showing the overlap between proteins that are altered, in any direction, in FUS^P525L^ motoneurons (“MASS-SPEC”) and transcripts that are bound in the 3′UTR by endogenous FUS^P525L^ (“3′UTR ENDO”) or exogenous FLAG-FUS^P525L^ (“3′UTR FLAG”). In (**d**, **e**), a red box indicates that the Fisher’s exact test p-values are considered significant. Specifically, *p* < 0.01 for the overlap between INTRON FLAG and MASS-SPEC in (**d**) and for the overlap between 3′UTR ENDO and MASS-SPEC in (**e**). Complete list of p-values are reported in Supplementary Fig. S2 online. The drawings have been taken by Maria Giovanna Garone.

Taken together, the proteomics analysis in FUS mutant human motoneurons revealed altered levels of factors involved in neuron development and catabolic processes and of proteins encoded by other ALS-related genes.

### Molecular mechanisms underlying proteome alteration in FUS mutant motoneurons

In order to get insights into the molecular basis of motoneuronal proteome alteration downstream of FUS mutation, we crossed the lists of differentially expressed mRNA transcripts^11^ and proteins (present work). Figure 2a shows the differentially expressed proteins, ordered by fold change, whose mRNA levels changed in either direction. For these genes we could not detect a robust correlation between transcript and protein levels (PCC = 0.342, *p* = 0.0287) (Fig. 2b), suggesting that, for most genes, the alteration of protein levels cannot be simply explained by changes of transcript levels. We next focused on known and predicted targets of the microRNA miR-375, which is downregulated in FUS^P525L^ motoneurons^11^. Indeed, six targets were significantly upregulated (VAT1L, DESI1, CTBP2, ARHGAP35, JUND and ACSL3; Fig. 2c), suggesting that miR-375 impairment might explain a small subset of altered proteins. Also in this case, we did not observe correlation between transcript and protein levels (PCC = 0.274, *p* = 0.599; Supplementary Fig. S1 online). Finally, we interrogated our dataset generated by photoactivatable ribonucleoside-enhanced crosslinking and immunoprecipitation (PAR-CLIP) analysis^12^. Specifically, in that work we identified transcripts bound by both endogenous and FLAG-tagged transgenic FUS, finding that FUS^WT^ preferentially binds intronic regions, while FUS^P525L^ is mostly associated with 3′UTRs. We reasoned that altered protein levels might result: (a) from transcripts selectively bound by FUS^WT^ in introns, as a consequence of decreased nuclear FUS in mutant cells; (b) from transcripts bound by the mutant protein in their 3′UTR, due to gain of aberrant functions of FUS^P525L^, presumably in the cytoplasm. We considered targets that overlapped between the endogenous and exogenous FUS datasets to be high confidence targets. Several transcripts, which are bound in an intron preferentially by FUS^WT^, showed altered protein levels in any direction (60/323; 18.6%; Fig. 2d; Supplementary Fig. S2 online). Notably, a large fraction of altered proteins is encoded by transcripts selectively bound by mutant FUS in the 3′UTR (216/323, 66.9%; Fig. 2e; Supplementary Fig. S2 online). We performed GO term enrichment analysis of this latter group of genes and noticed terms related to “RNA binding” and “Protein binding” molecular functions for both upregulated and downregulated proteins (Supplementary Table S3 online).

Together with our previous work, in which we showed aberrantly increased protein levels of some FUS^P525L^ targets encoding for RBPs^12^, these results suggest that the most likely mechanism underlying proteome alteration in mutant FUS motoneurons is aberrant targeting of 3′UTRs by mutant FUS.

### Mutant FUS alters protein levels of selected candidates via 3′UTR binding

**Figure 3:**
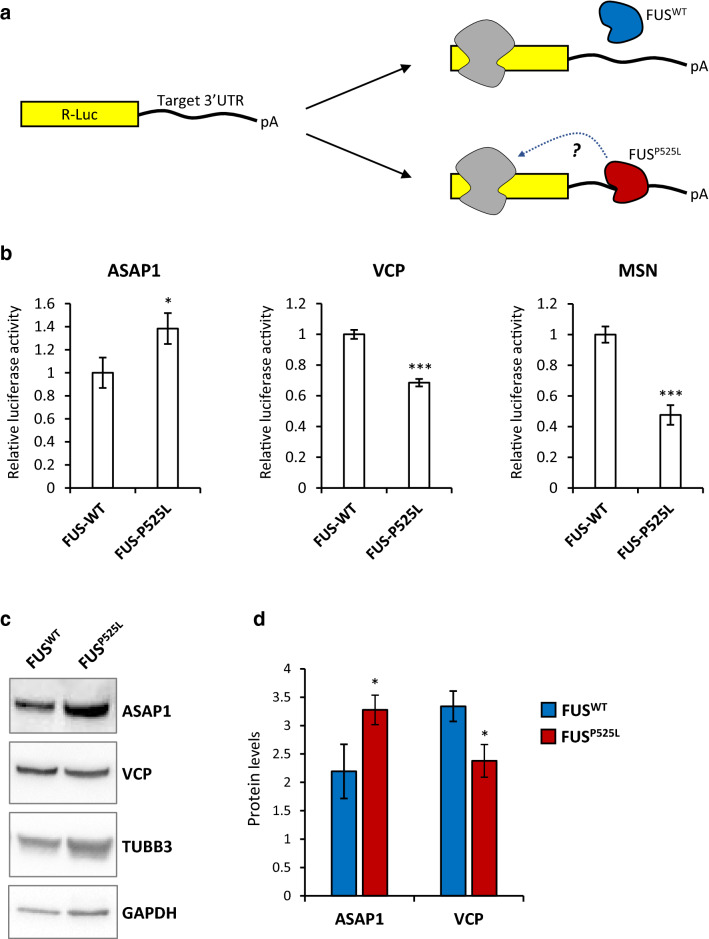
Candidate targets validation. (**a**) Schematic representation of the luciferase reporter assay used for validation of FUS^P525L^ regulation of protein levels via 3′UTR binding. (**b**) Luciferase assay on HeLa cells expressing RFP-FUS-WT and RFP-FUS-P525L and transfected with a luciferase construct containing the 3′UTR of the indicated candidates. Histogram bars represent the average of 3 experiments and error bars indicate the standard deviation (Student’s t test; paired; two tails; *p < 0.05; ***p < 0.001). Values have been normalized to the average of FUS-WT set as 1. (**c**) Western blot analysis of selected targets (ASAP1; VCP) and controls (neuronal tubulin beta 3, TUBB3; GAPDH) in hiPSC-derived motoneurons. (**d**) Quantification of Western blot signals of the indicated candidates. TUBB3 signal was used for normalization. Histogram bars represent the average of 3 independent differentiation experiments (shown in Supplementary Fig. S3 online) and error bars indicate the standard deviation (Student’s *t* test; paired; two tails; *p < 0.05).

We next aimed to experimentally validate the hypothesis that 3′UTR binding could alter translation of mutant FUS targets. A subset of genes showing altered protein levels and selective 3′UTR binding by FUS^P525L^ is shown in Supplementary Fig. S2 online. Candidates were primarily chosen for their link to neurodegenerative diseases, including ALS. On the basis of the GO analysis (Fig. 1b), we also included genes involved in neuron development and cytoskeleton dynamics. Collectively, we took into consideration 5 genes encoding for downregulated proteins (KIF5C, ARCN1, VAMP2, VCP and MSN) and 2 genes encoding for upregulated proteins (EZR and ASAP1). Reporter constructs were generated by fusing the candidate gene 3′UTR to the Renilla luciferase coding sequence and transfected into HeLa cells expressing WT or mutant FUS transgenes (Fig. 3a; Supplementary Fig. S3 online). The reporter fused to the *EZR* or *VAMP2* 3′UTR was not significantly altered by mutant FUS, whereas the 3′UTR of *KIF5C* and *ARCN1* conferred a slight decrease in luciferase activity (Supplementary Fig. S3 online). A more significant alteration was detected for the 3′UTR of *ASAP1* (increased activity) and *VCP* and *MSN* (decreased activity) (Fig. 3b). Western blot validation in FUS mutant motoneurons revealed increased ASAP1 and decreased VCP levels (Fig. 3c, d; Supplementary Fig. S3 online), in agreement with proteomics and luciferase assay data.

Collectively, these results suggest that 3′UTR binding by mutant FUS might be sufficient, for at least a subset of its targets, to trigger up- or down-regulation of protein levels.

## Discussion

In this work we took advantage of pure populations of hiPSC-derived motoneurons to study the impact of a FUS mutation, linked to severe and juvenile ALS^15^, on the human motoneuron proteome. Interestingly, GO term enrichment analysis suggested that proteins involved in catabolic processes and oxidation–reduction are upregulated in FUS mutant cells. On the other hand, downregulated proteins are involved in neuron development and cytoskeleton organization. Notably, recent evidence points to a genetic association with ALS for proteins that have a role in cytoskeletal dynamics^1^. In particular, ALS-linked mutations have been reported in *DCTN1* (dynein-associated polypeptide)^16^, *PFN1* (profilin, an actin cytoskeleton regulator)^17^, *TUBA4A* (tubulin 4A)^18^ and the annexin family member *ANXA11*^19^. Moreover, the ALS-linked gene *KIF5A*^20^ and the related gene *KIF5C*, which are reduced in sporadic ALS Peripheral Blood Mononuclear Cells^21^, encode for kinesins. Here we report altered levels of DCTN1, ANXA5 (related to ANXA11) and KIF5 proteins in FUS mutant motoneurons.

FUS is a multifaceted RBP playing multiple roles in RNA metabolism. Here we propose that in ALS motoneurons mutant FUS aberrant 3′UTR binding might lead to altered protein levels. Future work will shed light on the molecular mechanisms underlying these effects. We validated selected targets by reporter assay and Western blot. One of such transcripts is *MSN,* a member of the ERM family encoding for cross-linkers between the plasma membrane and the actin cytoskeleton, which is altered in postmortem tissues of ALS and FTD patients^22^. Another novel mutant FUS target is *ASAP1*, involved in regulation of membrane trafficking and cytoskeleton remodeling^23^. Finally, *VCP* is a known ALS-linked gene involved in regulation of protein homeostasis and autophagy^24^. These results uncover a broad impact of mutant FUS on expression other ALS-linked genes, oxidation–reduction processes and motoneuron cytoskeleton.

## Methods

### Cell culture and differentiation

Generation of iPSC lines and maintenance conditions are described in Lenzi et al., 2015^10^. The motoneuron differentiation protocol is detailed in De Santis et al., 2017^11^ and depicted in Supplementary Fig. S1 online. In brief, cells were differentiated in N2B27 medium (50% DMEM/F12, Sigma-Aldrich; 50% Neurobasal, Thermo Fisher Scientific; 1 × N2, 1 × Glutamax, 1 × MEM Non-Essential Aminoacids, all from Thermo Fisher Scientific; 1 × MACS NeuroBrew-21, Miltenyi Biotec; 100 U/ml Penicillin + 100 μg/ml Streptomycin, Sigma Aldrich) supplemented with 1 μM all-trans retinoic acid (Sigma-Aldrich) and 1 μM SAG (Merck Millipore) for 12 days in the presence of 10 μM SB431542 and 100 nM LDN-193189 (both from Miltenyi Biotec) from day 0 to 6, and 5 μM DAPT and 4 μM SU-5402 (both from Sigma-Aldrich) from day 6 to 12. Cells were sorted at day 12–13 using a FACSAria III (BD Biosciences) and re-plated on poly-L-ornithine- and laminin-coated dishes (both from Sigma-Aldrich) in Neural Medium (N2B27 medium supplemented with 20 ng/ml BDNF, 10 ng/ml GDNF, 10 ng/ml CNTF, all from Peprotech; 200 ng/ml L-ascorbic acid, Sigma-Aldrich). 10 μM Y-27632 (Enzo Life Sciences) was added for the first 24 h. For Western blot analysis, spinal motoneurons were obtained from hiPSCs as described in De Santis et al.^25^.

HeLa cells were purchased from ATCC and maintained in DMEM-F12 supplemented with 10% FBS, 1 × Penicillin/Streptomycin (all from Sigma-Aldrich) and 1 × Glutamax (Thermo Fisher Scientific).

### Proteomics analysis

Chemicals and solvents for proteomics analysis were purchased from Sigma-Aldrich, unless otherwise indicated. The NanoAcquity LC system and trapping column were purchased from Waters (Milford, MA, USA). The 5,600 + TripleTof MS system, ProteinPilot and MarkerView software were purchased from SCIEX (Ontario, Canada).

For disulfide bonds reduction, 50 μg of proteins were incubated at 56 °C for 30 min with 10 μl of 100 mM DTT (dithiothreitol) in digestion buffer (50 mM NH_4_HCO_3_ in MilliQ water, pH 8). Cysteine residues were then alkylated with 30 μl of 100 mM IAA (iodoacetamide) in digestion buffer for 20 min at room temperature in the dark. Protein precipitation with cold acetone was performed at − 20 °C before overnight digestion with 1 μg of trypsin at 37 °C in 50 mM NH_4_HCO_3_ in MilliQ water, pH 8. Tryptic peptides were dried under vacuum and then dissolved in 150 μl of 3% acetonitrile (ACN) + 0.1% formic acid (FA) for LC–MS/MS analysis.

1.66 μg of tryptic peptides were used for protein quantification. A NanoAcquity chromatographic system and a TripleTof 5,600 + mass spectrometer equipped with a NanoSpray III ion source were used for LC–MS/MS analysis. Peptides were first desalted during the trapping phase on a 180 μm × 20 mm Acquity C18 column for 4 min at 4.0 μl/min flow rate (1% ACN + 0.1% FA) and then moved to a PicoFrit C18 column (75 μm × 25 cm, from NewObjective Inc., Woburn, MA, USA). The separated peptides were eluted at 300 nl/min with a 2 h gradient of ACN in water (3% to 45%, both eluents were added with 0.1% FA). After the elution of the peptides, the column was washed with 90% of ACN for 5 min and then re-equilibrated to 3% ACN for 18 min. The mass spectrometer parameters were set as follows: ion spray voltage: 2,500 V, spray gas 1: 10, curtain gas: 30, declustering potential: 80 V and source temperature: 90 °C.

For protein quantification, the mass spectrometer operated in positive ion mode for data-independent acquisition (DIA), following the SWATH protocol for label free proteomics^26^. SWATH acquisition was performed using 400–1,250 m*/z* range for precursors ions, with a variable window width from 7 to 50 Da. 100 consecutive SWATH experiments, each lasting 25 ms, within 100–1,500 m*/z* mass range were performed after a full range survey scan of 250 ms. DIA spectra were searched against the PanHuman ion library^27^, using only no-shared peptides. The following parameters were used: 90% minimum peptide confidence, 50 ppm maximum mass tolerance, 30 min maximum RT tolerance and 6 MRM transitions per peptide.

### Bioinformatics analysis

PAR-CLIP reads and transitions were derived from published data as described in De Santis et al., 2019^12^; only RATIO of transitions (T ≥ C) > 1 comparing FUS^P525L^ vs FUS^WT^ were considered for the comparison with the proteomics data. Paired sample t-test was used to detect differentially expressed proteins from mass-spectrometry analysis and significance level was set to a *p* value < 0.05. RNA-seq data was derived from De Santis et al.^11^. Pearson’s correlation was calculated between paired proteomic and RNA-seq and statistical significance was computed using ‘cor.test’ function in R package. Intersection between PAR-CLIP and proteomics datasets was performed considering only genes that were detected in both experiments (4,326); statistical significance of the overlap was calculated with Fisher’s exact test. Heatmap was computed using Pheatmap function in R package with median as clustering method.

### Plasmid construction and transfection and luciferase assay

The 3′UTRs of candidate genes were amplified from genomic DNA using the oligos listed in Supplementary Table S4 online and inserted in the pSI-Check2 vector downstream the hRluc coding sequence. Reporter constructs were then transfected in 5 × 10^4^ pre-seeded HeLa cells, stably transduced with inducible WT or P525L mutant FUS transgenes^12^, in a 24-well plate using Lipofectamine 2000 (Life Technologies) following manufacturer’s instructions. After 24 h, cells were harvested and Renilla and Firefly luciferase activities measured by Dual Glo luciferase assay (Promega).

### Western blot

Western blot analysis was carried out using anti-VCP (5) (sc-57492; Santa Cruz Biotechnology), anti-ASAP1 (orb420376; Biorbyt), anti-TUBB3 (T2200; Sigma-Aldrich), anti-GAPDH (MAB-10578; Immunological sciences) primary antibodies and donkey anti-mouse IgG (H + L) (IS20404; Immunological Science) and donkey anti-rabbit IgG (H + L) (IS20405; Immunological Science) secondary antibodies, as previously described^12^. Uncropped images of the blots are shown in Supplementary Fig. S4 online.

## Supplementary information

Supplementary Information 1.

Supplementary Information 2.

Supplementary Information 3.

Supplementary Information 4.

## Data Availability

The raw LC–MS/MS data generated during this study have been deposited to the ProteomeXchange Consortium via the PRIDE partner repository with the dataset identifier PXD019596. Previously published RNA-seq raw data^11^ used in this study have been deposited at the GEO (GEO: GSE94888). Previously published PAR-CLIP raw data^12^ used in this study have been deposited at the GEO (GEO: GSE118347). All other data generated or analyzed during this study are included in this published article and its Supplementary Information files online.
