# Supplementary material for: Proteomics analysis of FUS mutant human motoneurons reveals altered regulation of cytoskeleton and other ALS-linked proteins via 3′UTR binding
Source: Sci Rep. 2020 Jul 16;10:11827. doi: 10.1038/s41598-020-68794-6 (PMC7366621; doi:10.1038/s41598-020-68794-6)
Supplement: Supplementary file 1 — Supplementary Information 1. [file 41598_2020_68794_MOESM1_ESM.pdf]

SUPPLEMENTARY INFORMATION

Proteomics analysis of FUS mutant human motoneurons reveals altered regulation of cytoskeleton and other ALS-linked proteins via 3'UTR binding

Maria Giovanna Garone[1,§], Vincenzo Alfano[1,§,#], Beatrice Salvatori[2], Clarissa Braccia[3], Giovanna Peruzzi[2], Alessio Colantoni[1,2], Irene Bozzoni[1,2], Andrea Armirotti[4] and Alessandro Rosa[1,2,5,*]

1. Department of Biology and Biotechnology Charles Darwin, Sapienza University of Rome, P.le A. Moro 5, 00185 Rome, Italy
2. Center for Life Nano Science, Istituto Italiano di Tecnologia, Viale Regina Elena 291, 00161 Rome, Italy
3. D3 PharmaChemistry, Istituto Italiano di Tecnologia, Via Morego 30, 16163 Genoa, Italy
4. Analytical Chemistry Lab, Istituto Italiano di Tecnologia, via Morego 30, 16163, Genoa, Italy
5. Laboratory affiliated to Istituto Pasteur Italia-Fondazione Cenci Bolognetti – Department of Biology and Biotechnology Charles Darwin, Sapienza University of Rome, Viale Regina Elena 291, 00161 Rome, Italy

§ those authors equally contributed

# Present address: Cancer Research Center of Lyon (CRCL) UMR INSERM 1052-CNRS 5286 151 cours Albert Thomas, 69424 Lyon Cedex 03, France

* Corresponding author: alessandro.rosa@uniroma1.it; Tel: +39-0649255218

SUPPLEMENTARY FIGURES S1-S4
SUPPLEMENTARY TABLES S1-S3 LEGENDS
SUPPLEMENTARY TABLE S4

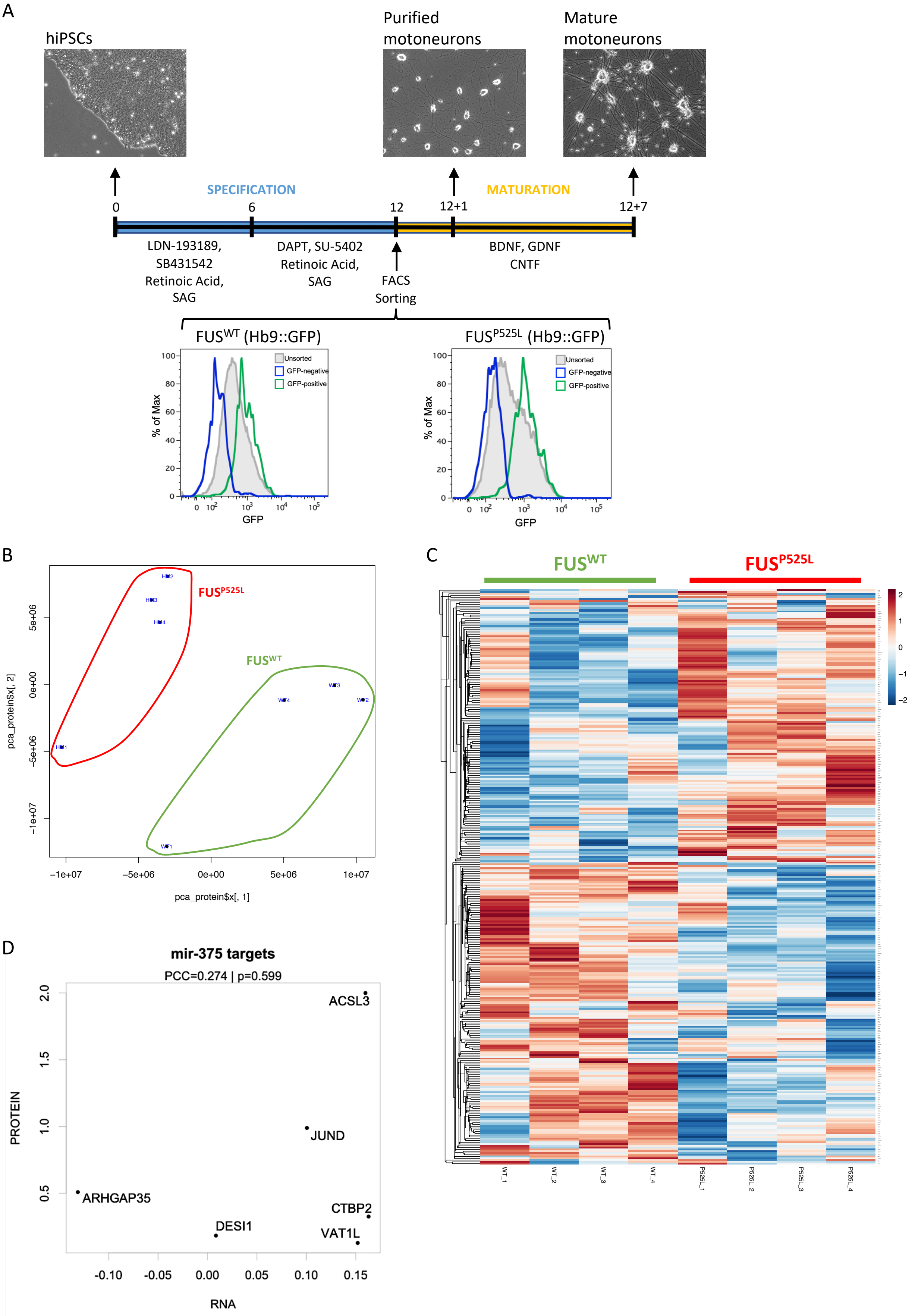
A
hiPSCs
Purified motoneurons
Mature motoneurons
SPECIFICATION
MATURATION
0
6
12
12+1
12+7
LDN-193189, SB431542 Retinoic Acid, SAG
DAPT, SU-5402 Retinoic Acid, SAG
FACS Sorting
BDNF, GDNF CNTF
FUSWT (Hb9::GFP)
FUSP525L (Hb9::GFP)
Unsorted
GFP-negative
GFP-positive
% of Max
GFP
B
FUSP525L
FUSWT
pca_protein$x[, 2]
pca_protein$x[, 1]
C
FUSWT
FUSP525L
WT_1
WT_2
WT_3
WT_4
P525L_1
P525L_2
P525L_3
P525L_4
D
mir-375 targets
PCC=0.274 | p=0.599
ACSL3
JUND
ARHGAP35
CTBP2
DESI1
VAT1L
PROTEIN
RNA

Supplementary Figure S1. Motoneuron differentiation protocol and proteomics analysis.

(A) Schematic of the motoneuron differentiation protocol, consisting in an initial specification phase and subsequent maturation. Under these conditions, the spinal motoneuron gene HB9 (MNX1) is expressed transiently at the end of the differentiation phase [11]. A Hb9::GFP reporter construct, stably integrated in the AAVS1 locus of both lines, allowed FACS-mediated isolation of pure populations of motoneurons. After sorting, GFP-positive and -negative cells were analyzed by FACS to check the purity of the sorted samples for FUS^{WT} and FUS^{P525L} samples. Representative plot overlays, shown here, confirm the purity of isolated populations compared to unsorted samples. (B) PCA plot showing clustering of FUS^{WT} (WT1-4) and FUS^{P525L} (HM1-4) motoneurons samples (4 independent differentiation experiments each). (C) Heatmap representing mass-spectrometry-derived levels of proteins that are differentially expressed between FUS^{WT} and FUS^{P525L} motoneurons Plotted values correspond to median-centered log2-transformed RPKM values. (D) Correlation between transcript and protein levels of genes enlisted in Figure 2C. PCC: Pearson correlation coefficient; p: p-value.

A

	MASS-SPEC	INTRON FLAG
INTRON ENDO	p=0.1405967	p=2.157536e-80
INTRON FLAG	p=0.001528546	
INTRON ENDO/FLAG	p=0.07559609	

B

	MASS-SPEC	3'UTR FLAG
3'UTR ENDO	p=0.003394452	p=8.6232e-282
3'UTR FLAG	p=0.3423602	
3'UTR ENDO/FLAG	p=0.08713203	

C

Gene name	PROT log2FC
KIF5C	-1,285821
ARCN1	-1,23009
VAMP2	-0,3774077
VCP	-0,1202776
MSN	-0,09434076
EZR	0,3203302
ASAP1	1,594612

D

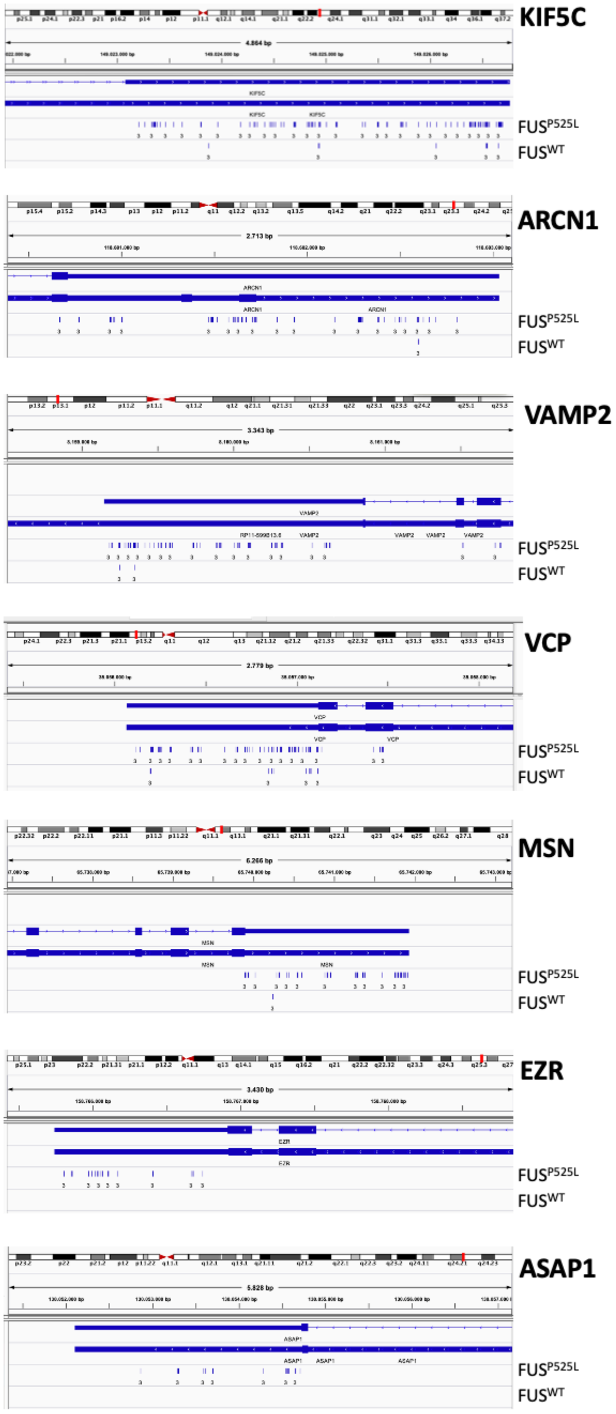

Supplementary Figure S2. Candidate genes from proteomics and PAR-CLIP.

(A) p-values relative to Figure 2D (Fisher's exact test). INTRON ENDO/FLAG indicates the overlap between INTRON ENDO and INTRON FLAG. (B) p-values relative to Figure 2E (Fisher's exact test). 3'UTR ENDO/FLAG indicates the overlap between 3'UTR ENDO and 3'UTR FLAG. (C) List of candidate genes analyzed in luciferase reporter assays shown in Figure 3. The fold change in proteins levels from the proteomics analysis in FUS^{WT} and FUS^{P525L} motoneurons is indicated. (D)

Snapshots of the Integrative Genomics Viewer (IGV) windows showing the mapping of PAR-CLIP reads and transitions (ref. [12]) on the loci encoding for the candidate genes.

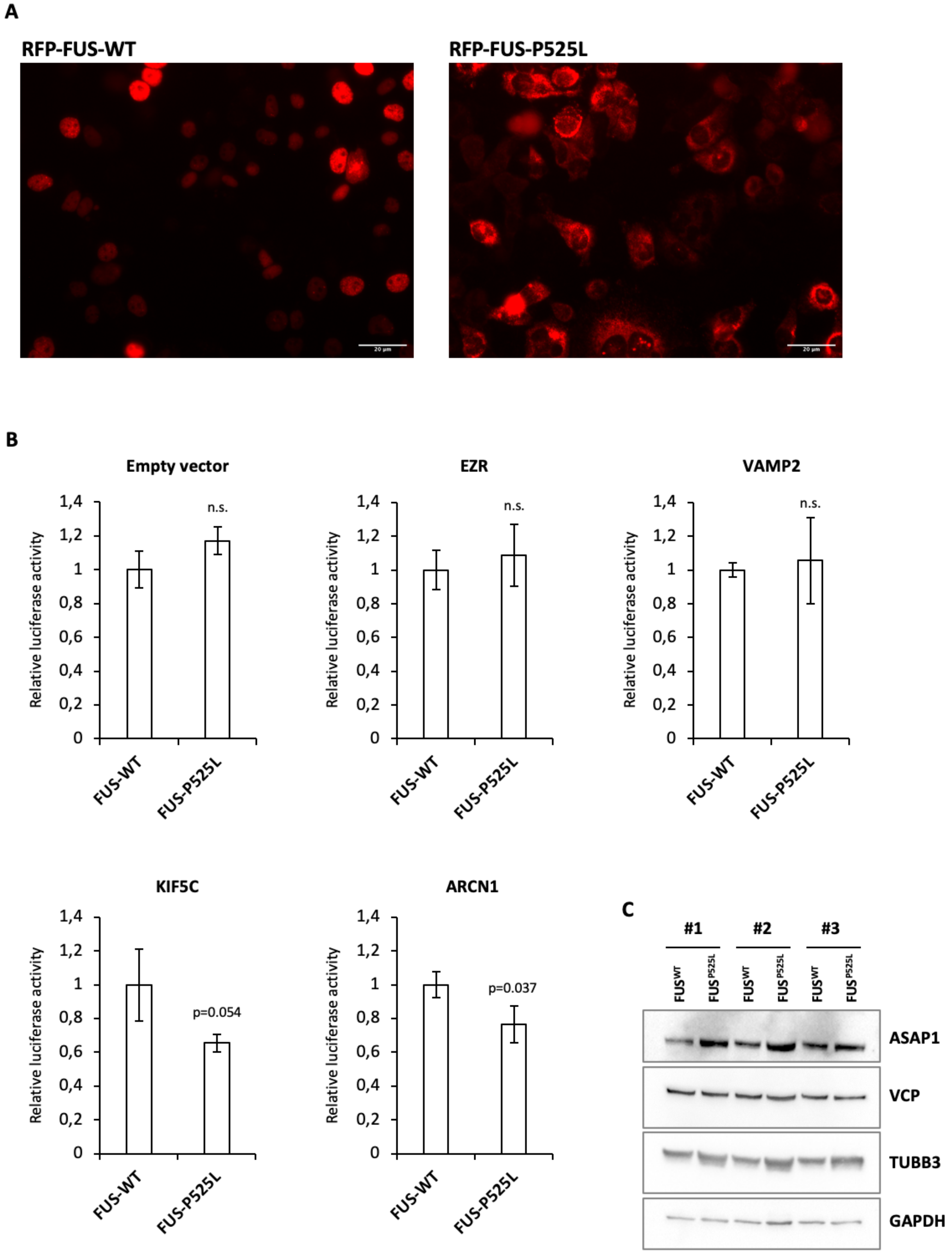

Supplementary Figure S3. Luciferase assay and western blot validation on candidate genes.

(A) HeLa cells expressing RFP-FUS-WT or RFP-FUS-P525L. Note nuclear localization of RFP-FUS-WT and partial cytoplasmic mislocalization of RFP-FUS-P525L. (B) Cells in (A) were

transfected with a luciferase construct devoid of 3'UTR (Empty vector) or containing the 3'UTR of the indicated candidates. Histogram bars represent the average of 3 luciferase assays and error bars indicate the standard deviation (Student's t test; paired; two tails; n.s.: not significant). Values have been normalized to the average of FUS-WT set as 1. (C) Replicates of the Western blot assay shown in Figure 3C-D and used for quantification.

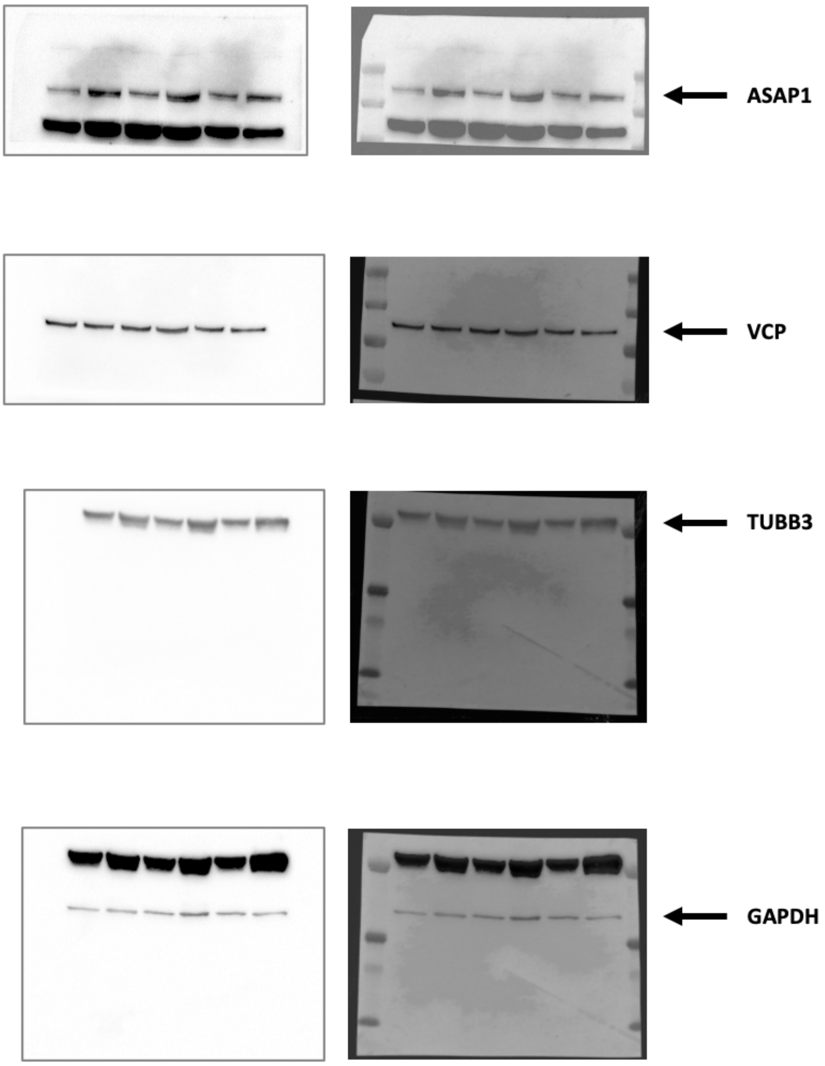

Supplementary Figure S4. Uncropped images of the western blots shown in Fig. 3 and Supplementary Figure S3C.

Panels on the right represent the overlap of the antibody signal and the molecular weight marker. The arrows indicate the expected size of the indicated proteins.

Supplementary Table S1. Genes encoding for differentially expressed proteins

This table reports data from RNA-seq experiment derived from De Santis et al., 2017 [11]: ensemble gene ID and gene symbol (Gene_ID, Gene_Name) with RPKM values from WT (RPKM_WT) and FUS^{P525L} (RPKM_MUT), log2 Fold change WT versus FUS^{P525L} (log2FC_RNA) and associated p. value; for the same gene we report proteomic analysis results from t-test log2 Fold Change WT versus FUS^{P525L} (log2FC_protein) and the associated p. value.

Supplementary Table S2. Differentially expressed genes in GO categories relative to Fig. 1B and 1C

This table reports Gene Ontology divided by categories of the upregulated and downregulated genes derived from proteomic analysis.

Supplementary Table S3. Differentially expressed genes in GO categories relative to Fig. 2E

This table shows an integrative Gene Ontology analysis of the genes that are: 1) found to be differentially expressed in proteomic analysis and 2) enriched for the binding of FUS^{WT} and FUS^{P525L} as identified from PAR-CLIP data. Among these genes, we selected the ones that are enriched in GO categories relevant for ALS pathophysiology and motor neuron degeneration (Selected terms_upregulated and Selected terms_downregulated).

**Gene name**	**3’UTR length**	**Forward primer**	**Reverse primer**
ARCN1	2286 bp	CACTCGAGTACCAAGAAGAGGGAGCTGA	CAGCGGCCGCATGTTAGGTCCCATTACAGC
ASAP1	2625 bp	CACTCGAGCAAAACGCAGAACCTTAAG	CAGCGGCCGCTGGGATCAACTGAGAATATG
EZR	1173 bp	CACTCGAGCAGCCAGGCCAGGACCAAGG	CAGCGGCCGCTTCGTGCCTTTGCAAAGC
KIF5C	3636 bp	CACTCGAGATATGACTCCACGTAGCATGT	CAGCGGCCGCTATACAACATGCAGTCTGCC
MSN	2054 bp	CACTCGAGTGGGCACCCAGCCTCTAGG	CAGCGGCCGCGAGTCACTGTGAAGTCTTCC
VAMP2	1708 bp	CACTCGAGATCCCCGAGGAGTCTGCCC	CAGCGGCCGCCTGGTTACTCATTACACAT
VCP	1002 bp	CACTCGAGGTGGTGGTGGCCAGCGTGCA	CAGCGGCCGCGGTGGACACAACTGTAAGTG

Supplementary Table S4. Primers used for 3’UTR cloning
